# *C. elegans* Enabled Exhibits Novel Interactions with N-WASP, Abl, and Cell-Cell Junctions

**DOI:** 10.1016/j.cub.2007.09.033

**Published:** 2007-10-23

**Authors:** Mark Sheffield, Timothy Loveless, Jeff Hardin, Jonathan Pettitt

**Affiliations:** 1 Program in Genetics, University of Wisconsin, 1117 West Johnson Street, Madison, Wisconsin 53706; 2 Cellular and Molecular Biology Graduate Program, University of Wisconsin, 1117 West Johnson Street, Madison, Wisconsin 53706; 3 Department of Zoology, University of Wisconsin, 1117 West Johnson Street, Madison, Wisconsin 53706; 4 School of Medical Sciences, University of Aberdeen Institute of Medical Sciences, Aberdeen AB25 2ZD, United Kingdom

**Keywords:** DEVBIO

## Abstract

Ena/VASP proteins are associated with cell-cell junctions in cultured mammalian cells [1] and *Drosophila* epithelia [2, 3], but they have only been extensively studied at the leading edges of migratory fibroblasts, where they modulate the protrusion of the leading edge [4]. They act by regulating actin-filament geometry, antagonizing the effects of actin-capping protein [5]. Embryos lacking the *C. elegans* Ena/VASP, UNC-34, display subtle defects in the leading edges of migrating epidermal cells but undergo normal epidermal morphogenesis. In contrast, embryos lacking both UNC-34 and the *C. elegans* N-WASP homolog have severe defects in epidermal morphogenesis, suggesting that they have parallel roles in coordinating cell behavior. GFP-tagged UNC-34 localizes to the leading edges of migrating epidermal cells, becoming redistributed to new junctions that form during epidermal-sheet sealing. Consistent with this, UNC-34 contributes to the formation of cadherin-based junctions. The junctional localization of UNC-34 is independent of proteins involved in Ena/VASP localization in other experimental systems; instead, junctional distribution depends upon the junctional protein AJM-1. We also show that Abelson tyrosine kinase, a major regulator of Enabled in *Drosophila*, is not required for UNC-34/Ena function in epithelia. Instead, our data suggest that Abelson kinase acts in parallel to UNC-34/Ena, antagonizing its function.

## Results and Discussion

### UNC-34 and WSP-1 Are Required for Dynamic Protrusive Activity

**Figure 1:**
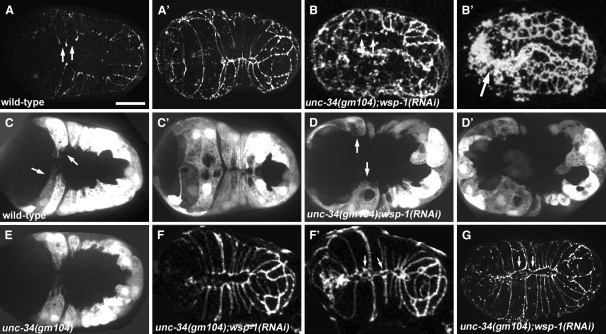
UNC-34 and WSP-1 Act Redundantly to Fulfill an Essential Role during Ventral Enclosure Projected confocal or multiphoton Z series, ventral views, with anterior to the left. (A)–(B′) and (F)–(G) show AJM-1::GFP in living embryos. (C)–(E) show DLG-1Δ7::GFP in living embryos. (F)–(G) show *unc-34(gm104);wsp-1(RNAi)* embryos with weaker enclosure phenotypes. The scale bar represents 10 μm. (A and A′) Wild-type enclosure. Leading cells (arrows) migrate ahead of posterior pocket cells (representative cells are marked with arrowheads). (B and B′) *unc-34(gm104);wsp-1(RNAi)* embryos fail to enclose properly. Leading cells ([B], arrows) show disrupted migration, and the epidermis ultimately retracts to the dorsal surface, ejecting pharyngeal tissue ([B′], arrow) and intestine to the surface of the embryo. (C and C′) Wild-type enclosure. Leading cells extend broad elaborate protrusions ([C], arrows) during migration toward the midline. (D and D′) *unc-34(gm104);wsp-1(RNAi)* embryos fail to enclose and have blunted leading cell protrusions ([D], arrows). (E) *unc-34(gm104)* embryos are not obviously disrupted, though they show subtle quantitative reduction in pocket cell protrusion dynamics. (F and F′) Embryos defective in cell migration and epithelial-sheet sealing, causing gaps to remain at the ventral midline junction ([F′], arrows). (G) Disrupted migration in ventral cells leads to hemi-junction formation (arrows).

Previous work showed that UNC-34/Enabled is not required for epidermal morphogenesis and that *unc-34* is genetically redundant with the gene encoding the sole *C. elegans* N-WASP homolog, *wsp-1*: Embryos lacking both proteins show defects in the ventral enclosure of the embryo [6]. We confirmed these results (Figures 1A–1B′; Movies S1 and S2 in the Supplemental Data available online) and examined genetic interactions between WVE-1/WAVE, WIP-1/WIP, and UNC-34 (Supplemental Results and Discussion). We further investigated the *wsp-1/unc-34* interaction by using dynamic analysis of protrusive activity. In wild-type embryos, the two pairs of ventral leading epidermal cells show extensive, relatively broad protrusions (Figure 1C). This protrusive region, which we refer to as the leading cell protrusive zone (LCPZ), has a dynamic perimeter and persists throughout migration (Movie S3). The pocket cells, in contrast, display much narrower projections (Figure 1C) whose lifetime is less than the 50 s interval we typically used for filming.

Protrusions in *unc-34(gm104); wsp-1(RNAi)* embryos appear to be quite different from those in the wild-type (Figures 1D and 1D′; Movie S4). Although the overall actin morphology is comparable to that of the wild-type (Figure S3), the LCPZ in these embryos is less dynamic, shorter, and blunted, and quantitative analysis shows that this difference is statistically significant (Wilcoxon rank sum test; α = 0.05; Supplemental Results and Discussion). Pocket cell migration is less disrupted, but the failure to complete enclosure causes the arrest of pocket cells before they reach the ventral surface of the embryo, making protrusions difficult to score. Although *unc-34* embryos enclose successfully and have normal LCPZ morphology (Figure 1E), pocket cell protrusions are somewhat shorter and are more infrequent than they are in the wild-type (α = 0.05; Supplemental Data). This phenotype is not scorable in *unc-34; wsp-1* embryos. We did not detect any such defects in the pocket cells of *wsp-1(RNAi)* embryos.

We attempted to test for epidermal-specific requirements for *wsp-1* and *unc-34* in several ways. First, we used an epidermal-specific RNA interference (RNAi) strain [7] to knock down *wsp-1* and *unc-34* function, but we observed no lethality, perhaps because the promoter used in this strain is active too late in development to confer robust RNAi sensitivity. Attempts to drive expression of *unc-34* in *unc-34(gm104)* mutants with a pan-epithelial promoter were likewise problematic because of selectively weak expression in the epidermis, although these results do suggest that at later stages, *unc-34* acts specifically in epithelia (see below). Finally, we used a strain designed to reduce *wsp-1* function in the epidermis, and this strain has been reported to result in some morphogenesis defects and lethality [8]. We could not replicate these results, nor have we ever observed similar effects when reducing *wsp-1* function alone in any other context, including the putative null deletion, *wsp-1(gm324)*. However, we did observe sporadic lethality in F_3_ embryos derived from *unc-34* homozygous mothers carrying the *wsp-1* knockdown constructs, with phenotypes qualitatively similar to *unc-34(gm104);wsp-1(RNAi)* embryos (data not shown). This suggests that *wsp-1* is specifically required in the epidermis. The synergistic genetic interaction between *unc-34* and *wsp-1* is consistent with several models at the molecular level (Figure S5).

### UNC-34 and WSP-1 Modulate Epidermal-Sheet Sealing

Actin-based protrusions are critical to cell migration, but they also contribute to epithelial-sheet sealing [1, 9]. Some *unc-34(gm104);wsp-1(RNAi)* embryos display weaker migration phenotypes, in which all cells eventually reach the midline (Figures 1F–1G). These embryos often display gaps between junctions at the midline (Figures 1F′ and 1G) and ultimately arrest because of rupture. Such phenotypes are consistent with the disruption of protrusions involved in junction formation. Moreover, in ventral cells that fail to meet at the midline, junctional molecules are recruited to the leading edge in the absence of contact with a contralateral partner (Figure 1G). This cell-contact-independent “hemi-junction” formation appears to be in response to a general apical junction development program involving both the DLG-1/AJM-1 complex (Figure 1G) and the cadherin complex (data not shown), and it occurs in other unrelated enclosure mutants and occasionally in wild-type cells (M.S. and J.H., unpublished data).

### UNC-34-GFP Localizes to the Leading Edge of Migrating Cells and to Apical Junctions

**Figure 2:**
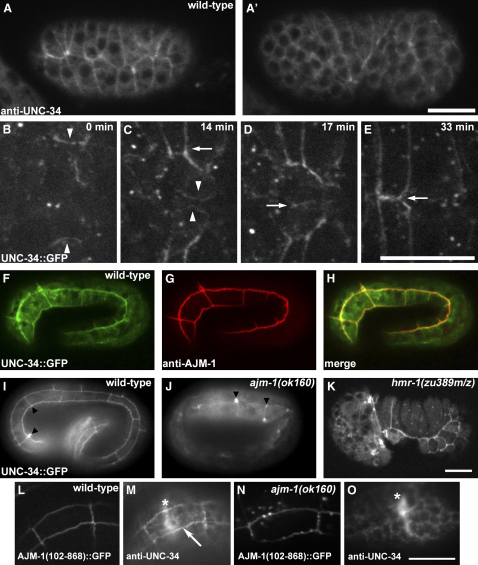
UNC-34 localizes to the leading edge of cell protrusions and to apical junctions. (A and A′) Confocal projections of dorsolateral (A) and ventrolateral (A′) surfaces (anterior is to the left) of the same embryo stained at mid-enclosure with anti-UNC-34 show broad expression throughout the embryo with enrichment at apical epidermal junctions. (B–E) Time-lapse sequence of projected confocal Z series, visualizing UNC-34::GFP in the ventral leading cell region (anterior is to the left) during enclosure. Besides apical junction enrichment, UNC-34::GFP is also present at the leading edge (arrowheads) of leading cell protrusions (C). Junctions enriched in UNC-34::GFP become apparent along the anterior and posterior borders of migrating cells ([C], arrow). Protrusions in contralateral partners meet at the ventral midline ([D], arrow), where apical junctions enriched with UNC-34::GFP eventually form ([E], arrow). (F–H) Confocal images of a wild-type 3-fold stage embryo expressing UNC-34::GFP (F) costained for AJM-1 (G). A merged image is shown in (H). (I–K) Representative wide-field images of wild-type (I), *ajm-1(ok160)* (J), and *hmr-1(zu389)* maternal and zygotic loss (K) embryos expressing UNC-34::GFP. UNC-34::GFP at apical junctions is misdistributed into puncta in *ajm-1* embryos ([J], arrowheads) but is largely normal in *hmr-1* embryos. (L–O) Immunostaining of UNC-34 in embryos carrying a truncated *ajm-1::gfp* transgene lacking a putative consensus binding site for Ena/VASP proteins (*ajm-1(102-868)::gfp*). (L) and (M) show a wild-type embryo expressing AJM-1(102-868)::GFP. UNC-34 localizes to junctions ([M], arrow). UNC-34 is also prominently expressed in neurons of the nerve ring (asterisk). (N) and (O) show an *ajm-1(ok160)* embryo rescued by *ajm-1(102-868)::gfp*. Although the truncated AJM-1 localizes to junctions (N), it is insufficient to localize UNC-34 there (O), despite robust expression in neurons (asterisk). Scale bars represent 10 μm.

A polyclonal antibody against UNC-34 shows broad cortical localization, as well as enrichment near apical junctions in epithelial cells (Figures 2A and 2A′). To better determine the subcellular localization of UNC-34 in living embryos, we generated a strain expressing full-length UNC-34 protein, tagged with green fluorescent protein (GFP) at its C terminus, under the control of the HMR-1A promoter (P_HMR-1A_). This promoter is active in all major epithelia during embryonic and postembryonic development, as well as in an undefined set of neurons.

GFP expression is first detected throughout the cytoplasm of most cells in early embryos. Once the epidermis is formed, UNC-34::GFP becomes enriched at cell-cell junctions. As leading cells approach the ventral midline, UNC-34 accumulates at the leading edge (Figures 2B and 2C, Movie S5). UNC-34 is also present at the leading edge of migrating pocket cells, but at a lower level. As morphogenesis proceeds, the junctional localization becomes more pronounced, and it persists throughout subsequent development (Figures 2C–2E). It is tempting to speculate that the cessation of cell migration is functionally linked to the relocation of UNC-34 to nascent junctions and that the relocation of UNC-34 is part of a hierarchy of changes in actin dynamics that favor the formation of cell junctions rather than continued protrusion. UNC-34::GFP also surrounds apoptotic cells as they are engulfed by epidermal cells (Figure S6).

### AJM-1 Participates in UNC-34 Recruitment to the Apical Junction

In primary keratinocytes, cadherin-catenin function is required for the recruitment of Mena and VASP to cell junctions [1]. We therefore examined UNC-34::GFP distribution in offspring of *hmr-1* germline mosaics, which lack both maternal and zygotic HMR-1/E-cadherin. These embryos display junctional localization of UNC-34::GFP that is essentially indistinguishable from wild-type embryos (Figure 2K). We obtained similar results with *hmp-1*/α-catenin and *hmp-2*/β-catenin null mutants (data not shown). Thus, the recruitment of UNC-34::GFP to the apical junction is independent from the cadherin-catenin complex. Vinculin and zyxin have also been implicated as Ena/VASP recruitment factors [1, 10]. However, embryos lacking the only *C. elegans* zyxin family member show normal junctional localization of UNC-34::GFP (data not shown), and vinculin is not expressed in *C. elegans* epithelial cells [11].

Ena/VASP proteins are recruited to subcellular sites through their EVH1 domains, which bind specifically to the F/LPPPP motif [12]. We therefore examined known junctional proteins for such motifs. AJM-1, a coiled-coil protein with some similarity to the vertebrate protein tricohyalin [13], contains the sequence DLPPPP, strongly matching the consensus for EVH1-binding peptides [14] and also colocalizes with UNC-34::GFP (Figures 2F–2H), making it a good candidate. We examined the localization of UNC-34::GFP in embryos homozygous for an *ajm-1* null allele, *ok160*. Although UNC-34::GFP still localizes to junctions in *ajm-1(ok160)* homozygotes, the intensity of the junctional signal is greatly reduced relative to the wild-type (Figure 2J), and its distribution along junctions is nonuniform, with the majority of UNC-34::GFP being concentrated at tricellular junctions.

We next tested whether the region of AJM-1 that includes the DLPPPP motif is necessary to maintain UNC-34 distribution by using *ajm-1(ok160)* mutants rescued with a transgene that does not contain this region but is sufficient for rescue of essential AJM-1 functions [13]. In wild-type embryos carrying the transgene, UNC-34 localizes to junctions (Figures 2L and 2M). In rescued *ajm-1* null mutants, however, little or no UNC-34 is detectable at junctions via immunostaining, even though nonepithelial cell types express UNC-34 normally (Figures 2N and 2O). Thus, although it is not the only factor influencing the localization of UNC-34, AJM-1 is a major determinant of its recruitment to epidermal cell junctions. Ena/VASP proteins can directly bind F-actin [15], so UNC-34 might also be recruited to junctions through its association with actin filaments, accounting for its AJM-1-independent junctional localization.

### UNC-34 Contributes to Cadherin-Mediated Epidermal-Sheet Sealing

**Figure 3:**
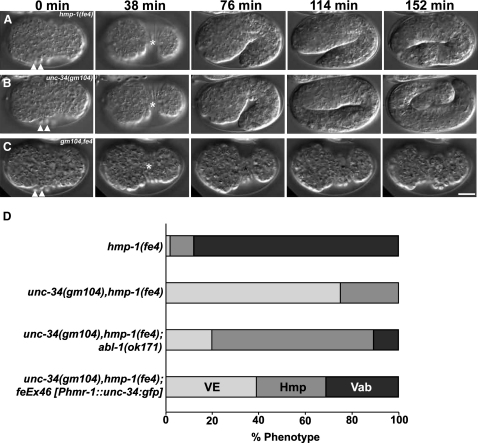
Loss of *unc-34* Function Enhances the Penetrance and Severity of *hmp-1(fe4)* Morphogenetic Defects (A–C) Time-lapse images showing the morphogenesis of representative embryos for each genotype: *hmp-1(fe4)* (A), *unc-34(gm104)* (B), and *unc-34(gm104) hmp-1(fe4)* double mutant (C). Anterior is to the left in all panels. The leading cells initiate ventral enclosure in all three embryos (arrowheads), but in *unc-34(gm104) hmp-1(fe4)* double mutants, the leading cells fail to establish junctions at the ventral midline. By 38 min, the *hmp-1(fe4)* and *unc-34(gm104)* embryos have completed ventral enclosure and begun elongation. Ventral enclosure is incomplete in the *unc-34(gm104) hmp-1(fe4)* embryo and the internal tissues are extruded by the contraction of the epidermis ([C], asterisk). Note that the *hmp-1(fe4)* embryo undergoes ventral enclosure at the same rate as the *unc-34(gm104)* embryo, but its elongation rate is reduced relative to *unc-34(gm104)*. Scale bar represents 10 μm. (D) Quantification of the morphological defects displayed by *hmp-1(fe4)* and *unc-34(gm104) hmp-1(fe4)* embryos. The bars show the proportion of embryos showing each class of defect. The following abbreviations are used: ventral enclosure defects (VE), embryos that arrest at less than twice the premorphogenetic length (Hmp), and embryos that elongate beyond 2-fold (Vab; this class is the only one to contain viable embryos able to reach fertile adulthood). At least 500 embryos were scored for each genotype.

In order to further investigate the role of UNC-34 at epithelial junctions, we took advantage of a hypomorphic mutation affecting the *C. elegans* α-catenin, HMP-1, which sensitizes cells to perturbations in cadherin function [16]. We constructed double-mutant combinations between *hmp-1(fe4)* and *unc-34(gm104)*. *hmp-1(fe4)* mutants show variable defects in epidermal morphogenesis (Figures 3A and 3D): A small minority of animals arrest with defects in ventral enclosure, but the majority are viable and fertile. In contrast to the respective single mutant phenotypes (Figures 3A, 3B, and 3D), the *unc-34 hmp-1(fe4)* double-mutant combination exhibits completely penetrant maternal-effect lethality (Figures 3C and 3D). Significant numbers of arrested embryos display defects in ventral enclosure indicative of embryos lacking cadherin function. Embryos that successfully enclose display severe elongation defects (Hmp).

To investigate the basis of this lethality, we filmed *unc-34(gm104) hmp-1(fe4)* embryos. Of 15 embryos that subsequently failed to undergo ventral enclosure, four showed complete retraction of the leading edge of the ventral epidermis dorsally and 11 showed partial ventral enclosure but extruded their internal organs once elongation began (Figure 3C). In all cases, the leading cells migrated to the ventral midline; the defect is therefore likely due to the failure to establish stable junctions, rather than a prior failure in leading cell migration. This defect is characteristic of animals completely lacking cadherin-catenin function [9]. The *unc-34::gfp* transgene complemented the embryonic lethality of *unc-34 hmp-1(fe4)* double mutants (Figure 3D).

We next examined the localization of HMR-1 in *gm104 fe4* double-mutant embryos. The distribution of HMR-1 is abnormal in *hmp-1(fe4)* embryos, showing occasional punctate accumulations and discontinuities [16]. However, there was no detectable difference in the distribution of HMR-1 in *gm104 fe4* double mutants compared to either single mutant (Figure S7). Thus, the enhanced morphogenetic defects caused by the loss of *unc-34* function are not attributable to loss of HMR-1/cadherin localization.

To determine whether *unc-34/hmp-1(fe4)* synergy occurs specifically in epithelial cells, we used the *dlg-1* promoter to drive *unc-34*::*gfp* expression in epithelial cells (see Supplemental Experimental Procedures). This construct is weakly and incompletely expressed in the epidermis (J.P., unpublished data), but we observed partial rescue of the synthetic lethality in the brood of an *unc-34(gm104) hmp-1(fe4)* homozygote expressing *unc-34*::*gfp*. This result suggests that the loss of *unc-34* function in epithelia is responsible for the observed synthetic lethality.

Recent work has led to the proposal that α-catenin acts to regulate actin dynamics at nascent adherens junctions, favoring the formation of unbranched, bundled actin filaments, and suppressing branched actin networks (reviewed in [17]). This would explain the requirement for UNC-34 in animals with impaired HMP-1 function; in this case, UNC-34 might partially compensate for reduced α-catenin activity. Given the association of Ena/VASP proteins with adherens junctions in other organisms, it is likely that there is a conserved relationship between α-catenin and Ena/VASP proteins.

### Abelson Tyrosine Kinase Antagonizes UNC-34 Function at Cadherin-Based Junctions

*Drosophila* Abelson tyrosine kinase (Abl) regulates epithelial morphogenesis, at least in part by regulating the localization of Enabled [3, 18]. We thus examined the role of the single *C. elegans* Abl homolog, ABL-1. A putative null allele, *abl-1(ok171)*, displays no obvious defects in epidermal morphogenesis [19], and *hmp-1(fe4)*; *abl-1(ok171)* double mutants show no enhancement of the *hmp-1(fe4)* mutant phenotype. We examined the effect of the *abl-1(ok171)* mutation on *unc-34(gm104) hmp-1(fe4)* synthetic lethality by constructing *unc-34(gm104) hmp-1(fe4)*; *abl-1(ok171)* triple-mutant homozygotes. The loss of *abl-1* function partially suppressed the morphogenetic defects observed in *unc-34 fe4* double mutants (Figure 3D); a minority of embryos were able to undergo elongation beyond twice their premorphogenetic length, and some hatched and developed into fertile adults. Thus, the loss of *abl-1* function partially alleviates the requirement for *unc-34* function in *hmp-1(fe4)* embryos. Because *unc-34(gm104)* does not produce any detectable UNC-34 protein, *abl-1(ok171)* appears to act as a bypass suppressor, a genetic interaction that implies that unlike in *Drosophila*, Abelson kinase acts in parallel to UNC-34/Ena and that it antagonizes UNC-34 function.

In vitro experiments indicate that Ena/VASP proteins antagonize capping protein [5]; *abl-1* loss of function could lead to a reduction of actin-capping activity and thereby partially relieve the requirement for UNC-34. In order to address this possibility, we attempted to suppress the synthetic lethality of *unc-34(gm104) hmp1(fe4)* by reducing the expression of the two *C. elegans* capping proteins with RNAi. However, even a mild reduction in the expression of these proteins resulted in arrest during early embryonic development, precluding assessment of genetic suppression (data not shown).

In summary, our studies have uncovered novel interactions between UNC-34/Ena and components of both the leading edge and cell-cell junctions in the *C. elegans* epidermis. Future work aimed at identifying molecular components that recruit and modulate UNC-34/Ena function in these subcellular compartments should yield further insights into how this important actin regulator functions during morphogenesis.

## Note Added in Proof

While this work was under review, Gates et al. published a report describing the requirement for and dynamic localization of Ena during morphogenesis in the *Drosophila* embryo [20]. They found that Ena is dispensable for dorsal closure and for general cell-cell adhesion but that Ena modulates protrusive activity at the leading edge of the epidermis, where it localizes to filopodia. This work is consistent with our analysis of lamellipodial localization of UNC-34 during ventral enclosure and with our analysis of roles for UNC-34 during cadherin-mediated events in *C. elegans*.

## Supplemental Data

Document S1. Supplemental Results and Discussion, Experimental Procedures, and Seven Figures

Movie S1. Ventral Enclosure in the Wild-Type Visualized with AJM-1::GFP A projection of from a 4D multiphoton movie of an embryo expressing *ajm-1::gfp* (ventral view, anterior is to the left). The interval between frames is 180 s.

Movie S2. Morphogenesis Defects in an *unc-34(gm104);wsp-1(RNAi)* Embryo Visualized with AJM-1::GFP Dorsal (right) and ventral (left) projected views of the same *unc-34(gm104);wsp-1(RNAi)* embryo from a 4D multiphoton movie of an embryo expressing *ajm-1::gfp* (anterior is to the left in each case). The interval between frames is 180 s.

Movie S3. Leading Cell Protrusive Zone Activity in the Wild-Type Visualized with a *dlg-1* Transcriptional Reporter Projections of *dlg-1Δ7::gfp* expression from a spinning-disc confocal 4D movie (ventral view, anterior is to the upper left). The interval between frames is 50 s.

Movie S4. LCPZ activity in an *unc-34(gm104);wsp-1(RNAi)* Embryo Visualized with a *dlg-1* Transcriptional Reporter Projections of *dlg-1Δ7::gfp* expression from a spinning-disc confocal 4D movie (ventral view, anterior is to the upper left). The interval between frames is 50 s.

Movie S5. UNC-34::GFP Localization in the Wild-Type during Ventral Enclosure Projections from a spinning-disc confocal 4D movie (ventral view, anterior is to the left). The interval between frames is 20 s.
