# Supplementary material for: C. elegans Enabled Exhibits Novel Interactions with N-WASP, Abl, and Cell-Cell Junctions
Source: Curr Biol. 2007 Oct 23;17(20):1791–6. doi: 10.1016/j.cub.2007.09.033 (PMC2045632; doi:10.1016/j.cub.2007.09.033)
Supplement: Document S1. Supplemental Results and Discussion, Experimental Procedures, and Seven Figures [file mmc1.pdf]

Supplemental Data

C. elegans Enabled Exhibits Novel Interactions with N-WASP, Abl, and Cell-Cell Junctions

Mark Sheffield, Timothy Loveless, Jeff Hardin, and Jonathan Pettitt

Supplemental Results and Discussion

UNC-34 Aids *Ce*SCAR/WAVE in Initiating Cell Migration

Several barbed-end modulators act via the Arp2/3 complex, which nucleates new actin filaments via the formation of side branches from existing filaments [S1]. Reducing the function of various members of the Arp2/3 complex results in embryonic lethality in *C. elegans* well before the initiation of morphogenesis [S2]. However, in order to perform its role as an actin nucleator, Arp2/3 must be bound and activated by one of several families of molecules, allowing for the localized activation of Arp2/3 in specific places and at specific times during development.

The SCAR/WAVE family of molecules is one such family of Arp2/3 activators [S3]. SCAR/WAVE forms part of a polypeptide complex that responds to activated Racs, or other membrane-associated signals, which in turn activates Arp2/3, thereby promoting actin-filament nucleation [S4–S7]. The *C. elegans* genome contains one predicted member of this family, *wve-1* [S8]. *wve-1(RNAi)* results in embryonic lethality (75.6%, n = 1018), with affected embryos displaying severe defects in embryogenesis (Figure S1B). *wve-1(RNAi)* does not affect the formation of the epidermis, but cells fail to perform the migrations necessary for proper dorsal intercalation and enclosure. Embryos ultimately arrest without completing morphogenesis with a terminal phenotype identical to Gex (gut on the exterior) mutants [S9].

Performing *wve-1* RNAi in *unc-34(gm104)* animals yielded both moderately increased lethality (90.2% n = 112) and slightly more severe phenotypes. Epidermal cells appeared rounded and did not initiate cell migrations (Figure S1C). In *C. elegans*, the loss of function of only one Rac, *ced-10*, yields extensive embryonic defects [S10]. *ced-10(n3417)*, a maternal-effect allele, causes lethality (89.8%, n = 430) and phenotypes strikingly similar to those in *unc-34(gm104); wve-1(RNAi)* embryos (Figure S1D). These phenotypes suggest that WVE-1, perhaps augmented by UNC-34, mediates the bulk of Rac signaling to the actin cytoskeleton during morphogenesis.

Ena/VASP and WASP/WIP Act in Parallel during Ventral Enclosure

The WASP family of molecules constitutes another important group of Arp2/3 activators [S11]. This activation can occur by WASP binding to active Cdc42 and/or PIP_2 at the membrane, and it often leads to the formation of

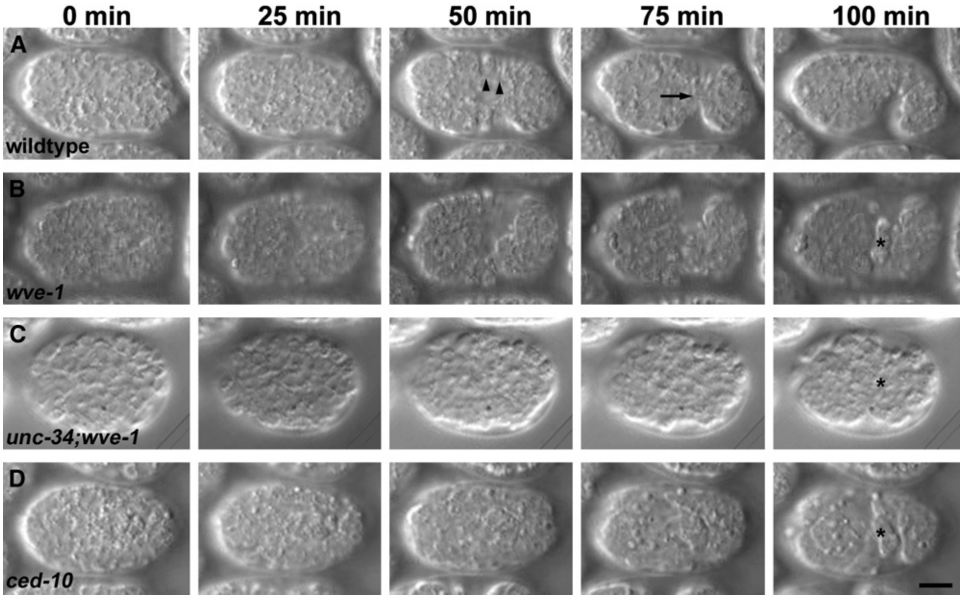

Figure S1. Initiation of Epidermal Migration Requires WVE-1 and UNC-34

Nomarski time-lapse images. Ventral is up, and anterior is to the left.

(A) Wild-type enclosure. Ventral cells migrate around the lateral surfaces (arrowheads) of the embryo to meet at the ventral midline (arrow).

(B) *wve-1(RNAi)* embryos in which epidermal cells fail to migrate and never enclose (asterisk).

(C) *unc-34(gm104); wve-1(RNAi)* embryos also fail during morphogenesis, with epidermal cells becoming slightly rounded and never migrating to enclose the animal (asterisk).

(D) *ced-10(n3417)* embryos phenocopy *unc-34(gm104); wve-1(RNAi)* embryos, including enclosure defects (asterisk).

The scale bar represents 10 μm.

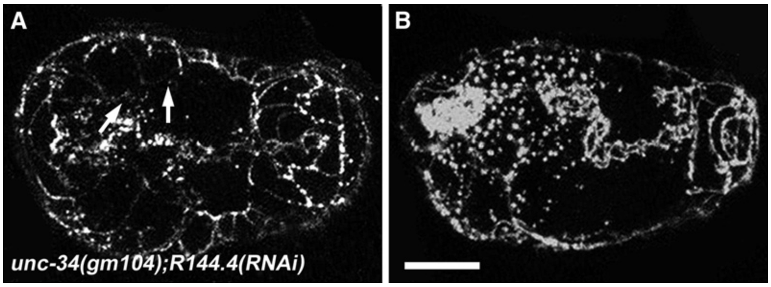

Figure S2. Loss of R144.4/WIP Enhances Migration Defects in *unc-34 (gm104)* Mutants

unc-34(gm104);R144.4(RNAi) phenocopies *unc-34(gm104);wsp-1(RNAi)*. Leading cells (arrows) show disrupted migration (A), and enclosure fails (B). The scale bar represents 10 μm.

filopodia [S12, S13]. In our hands, the removal of the function of the sole *C. elegans* N-WASP, *wsp-1*, via RNAi resulted in embryos that were largely phenotypically normal, with a low level of lethality (15.6%, n = 596); dead embryos displayed phenotypes consistent with disruptions in cell division (data not shown). However, as previously reported [S8], the reduction of WSP-1 in an *unc-34* null background yielded 100% lethality (n = 125); affected embryos displayed substantially disrupted morphogenesis (see Figure 1).

Because these defects are not observed in either single mutant, these results show that during morphogenesis, *unc-34* and *wsp-1* act in a genetically redundant manner. In order to support the genetic specificity of this interaction, we examined the role of the *C. elegans* WIP (WASP-interacting protein) during enclosure. The precise molecular role of WIP is unclear (reviewed in [S14]). It is capable of binding WASP family members [S15] and often appears to be important for WASP function [S16, S17], but it also participates in actin dynamics independent from WASP. *C. elegans* has a single WIP homolog, *wip-1* [S18]. In wild-type animals, *wip-1(RNAi)* results in low levels of embryonic lethality similar to *wsp-1 (RNAi)*, and double RNAi of *wsp-1* and *wip-1* does not enhance this lethality. *unc-34(gm104)*; *wip-1(RNAi)* and *unc-34(gm104); wsp-1(RNAi)* animals are phenotypically indistinguishable (Figures S2A and S2B; cf. Figures 1B and 1B′), strongly suggesting that *wip-1* and *wsp-1* act together during enclosure and that, in this context, WIP acts as a positive regulator of WASP function. One caveat to these experiments is that the RNAi knockdown for *wip-1* might have been incomplete. Future experiments involving *wsp-1* and *wip-1* null mutants would definitively confirm these results.

Overall Actin Morphology Is Grossly Normal in *unc-34(gm104);wsp-1(RNAi)* Embryos

The presumptive roles of UNC-34 and WSP-1 involve the modulation of the actin cytoskeleton. Actin is known to be generally required for ventral enclosure [S19], so we performed phalloidin staining to investigate the gross structure of actin in *unc-34;wsp-1* embryos (Figure S3). Phalloidin-stained *unc-34(gm104);wsp-1(RNAi)* embryos resemble wild-type embryos. Although leading-edge protrusions are less readily apparent, actin is enriched at the leading free edges and cell-cell borders of ventral cells that fail to migrate, and the bundling of cellular actin into nascent circumferential cables appears to initiate normally (Figure S3B).

Models for the Genetic Interaction between *unc-34* and *wsp-1*

Our results regarding the genetic interaction between *unc-34*/Ena and other components involved in leading-edge migration, collated with those of others, is shown in Figure S5A. The synergistic genetic interaction between *unc-34* and *wsp-1* is consistent with several models at the molecular level (Figure S5B). WSP-1 is known to activate the *C. elegans* Arp2/3 complex [S20] to promote actin-filament nucleation via side branching. UNC-34 presumably acts in a manner similar to other members of the Ena/VASP family because *unc-34* mutants display phenotypes [S8] that appear to be similar to neuronal phenotypes associated with the loss of Ena/VASP family proteins in other systems (reviewed in [S21]). One model of filopodium formation involves actin branches in the dendritic network becoming privileged via the presence of filament tip complexes that include Ena/VASP proteins [S22], which allow pre-existing filaments to elongate and ultimately become bundled into filopodia. So that redundancy between UNC-34 and WSP-1 can be integrated into this model, in *C. elegans*, most of the dendritic network could be generated via WVE-1-mediated branching. In addition, a local microenvironment with elevated WSP-1 activity—possibly because of active Cdc42 or another signal from the membrane—combined with UNC-34/tip complex activity could lead to a more elaborate protrusion structure.

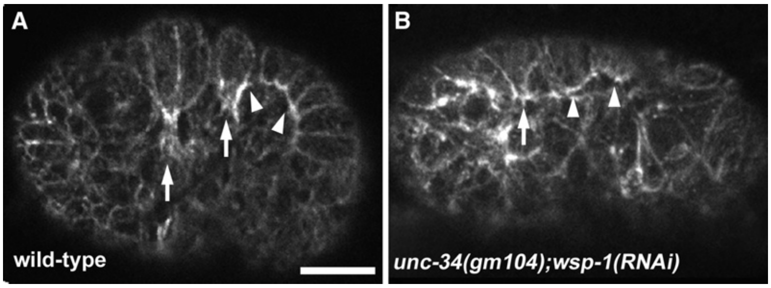

Figure S3. Phalloidin Staining of Wild-Type and *unc-34(gm104);wsp-1(RNAi)* Embryos during Ventral Enclosure

Phalloidin staining of fixed embryos.

(A) In the wild-type, actin is enriched at cell-cell borders, leading edges of migrating cells (representative arrowheads), and in epidermal cell protrusions (arrows).

(B) *unc-34(gm104);wsp-1(RNAi)* embryos show grossly normal actin structure, including enriched actin at leading edge (arrowheads), small protrusions (arrow), and cell-cell borders, as well as initial aggregation of bundled actin that will ultimately become circumferential bundles.

The scale bar represents 10 μm.

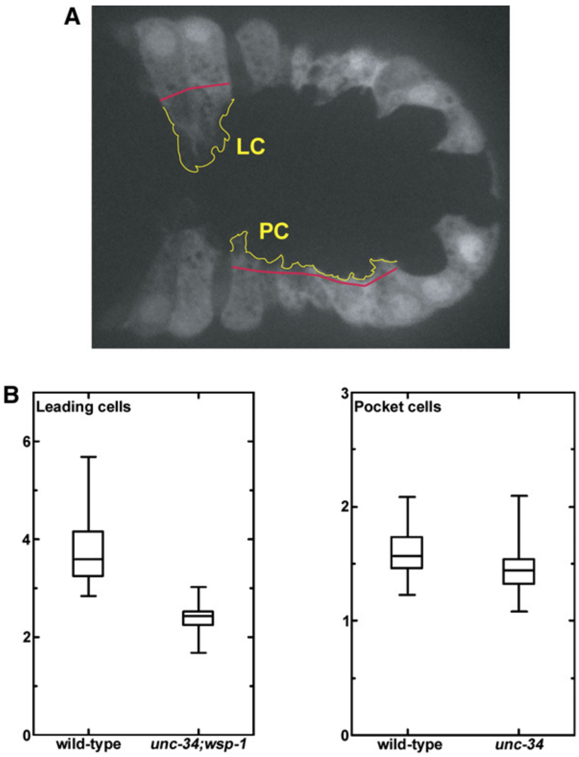

Figure S4. Quantification of Protrusive Activity

(A) Quantification of leading cell (LC) and pocket cell (PC) protrusions is performed by the division of the protrusion perimeter (yellow curves) by cell width (red lines).

(B) Left: Box and whisker plots of quartiles for leading cell protrusion perimeter divided by the width of cell bodies in wild-type and *unc-34(gm104);wsp-1(RNAi)* embryos show sample spread, median values, and interquartile range. Values for mutant embryos are distinct from those of the wild-type, as confirmed by Wilcoxon rank sums ($\alpha = 0.05$). Right: Box plots of quartiles for pocket cell protrusion perimeter divided by the width of cell bodies in wild-type and *unc-34(gm104)* embryos. Values for mutant embryos are distinct from those of the wild-type, as confirmed by Wilcoxon rank sums ($\alpha = 0.05$).

In such a growing-end model (Figure S5B, left), a certain critical threshold level of available barbed ends must be achieved through the activity of both UNC-34 and WSP-1, but each molecule is dispensable on its own. The loss of both molecules simultaneously is clearly catastrophic for protrusion formation.

An alternative, but not mutually exclusive, de novo-nucleation model (Figure S5B, right) offers a slightly different explanation for our results. Recent work in *Dictyostelium* has shown an interaction between the formin dDia2 and an Ena/VASP family protein during filopodium formation [S23]. If UNC-34 acts as a scaffolding molecule linking a formin (or other nucleator) to the existing cytoskeleton, it becomes easier to understand the genetic interaction of *wsp-1* and *unc-34*. In this case, WSP-1 provides one method of de novo filament nucleation, and UNC-34 provides another by recruiting a formin, with the two methods being functionally redundant in this context. The examination of the fine structure of the actin cytoskeleton in *unc-34; wsp-1* embryos, or their equivalents in another system, would no doubt provide insight into the molecular nature of this genetic interaction.

Supplemental Experimental Procedures

Strains

The N2 Bristol strain was used as wild-type and was the basis for all strains. The following mutations were used: *hmr-1(zu389)* I [S24], *unc-34(e315, e566, gm104)* V [S8], *hmp-1(zu278, fe4)* V [S24, S25], *abl-1(ok171)* X [S26], and *ajm-1(ok160)* X [S27]. The *mIs10* chromosome V rearrangement (an integrated transgenic array consisting of the *myo-2::GFP*, *pes-10::gfp* and *F22B7.9::gfp* transgenes), which suppresses recombination over a large region of LG V, was used for the balancing of *unc-34,hmp-1(fe4)* double-mutant chromosomes. *hT2[bli-4(e937) let(q782) qIs48]* [S28] was used for the balancing of *hmr-1(zu389)*. SU93 contains an integrated array (*jcIs1*) on linkage group IV that includes a truncated *ajm-1::gfp* encoding amino acids 102–868 of the predicted protein [S27]. SU159 carries the same transgene carried as an extrachromomal array in an *ajm-1(ok160)* null mutant background [S27]. Strain SU166 includes *jcIs1* and *unc-34(gm104) V*. SU220 has an extrachromosomal array (*jcEx60*) including *dlg-1Δ7::gfp* [S29]. SU226 is *unc-34(gm104)* V, and carries *jcEx60*. *jcEx60* was subsequently integrated, and the strain was outcrossed to make strain SU276. Strain LE179 carrying *ced-10(n3417) / lin-1(e1275ts),dpy-13(e184)* IV was a gift from Erik Lundquist.

So that germline mosaics of *hmr-1(zu389)* and *hmp-1(zu278)* could be obtained, individual adult hermaphrodites derived from transgenic lines carrying extrachromosomal arrays containing wild-type *hmr-1* or *hmp-1* genes, respectively, were picked to separate plates, and their progeny were examined for 100% embryonic arrest. Animals with this phenotype were placed on fresh plates without food for approximately 16 hr, and their broods were processed for immunostaining.

Generation of *unc-34::gfp* Transgenic Lines

The HMR-1A promoter (P_{HMR-1A}) was fused to the *unc-34* gene via PCR fusion, and the resultant amplicon was fused to the *gfp* gene and *unc-54* 3′ untranslated region (UTR) via a second polymerase chain reaction (PCR) fusion reaction. A list of the primer sequences used to generate the fusion amplicons is available on request to J.P. The P_{HMR-1A}::*unc-34*::*gfp* fusion amplicon was coinjected along with the *rol-6(su1006)* marker gene into *unc-34(gm104) hmp-1(fe4)/mIs10* hermaphrodites, and three independent lines carrying both transgenes as an extrachromosomal array were established. All three gave identical patterns of expression.

To express *unc-34::gfp* under the control of the *dlg-1* promoter, the upstream region of *dlg-1* was amplified from wild-type DNA and cloned into a modified version of pPD95.75 (provided by A. Fire, Stanford University), which can be used as a destination vector in the Gateway cloning system (Invitrogen). The primers used to amplify the *dlg-1* upstream region were dlg1PL, 5′-CGAAGCTTCA CAGTTTACCAAACTAGTC-3′, and dlg1PR, 5′-CGGCATGCGCTTCC TTCCTTCGGTG-3′. The *unc-34* cDNA was then cloned into this vector via recombination. This construct produced a fusion of the GFP gene to the C terminus of UNC-34. The resultant vector, pPE#JP101 was injected into *unc-34(gm104) hmp-1(fe4)/mIs10* hermaphrodites.

RNAi

Double stranded RNA-mediated interference was performed as previously described via either injection [S30] or feeding [S31] methods. Ambion MegaScript T7, T3, and SP6 kits were used for the preparation of injection samples. Plasmid pBG1 (described in [S8]) is a partial *wsp-1* cDNA cloned into Fire vector kit plasmid L4440 for feeding RNAi. *R144.4* RNA was made with partial cDNA yk433c4, from the laboratory of Yuji Kohara, as a template. *wve-1* RNA was made with a genomic fragment amplified with primers 5′-AAGTTAAGCT GAAGCCGAGAGCCTGCGG-3′ and 5′-GAACATCTTCGGAGAGATT TATCACGACG-3′ as the template.

Microscopy

4D Nomarski microscopy was performed as previously described [S32]. In brief, gravid adults were bisected in M9 solution so that embryos could be collected. Embryos were transferred by mouth

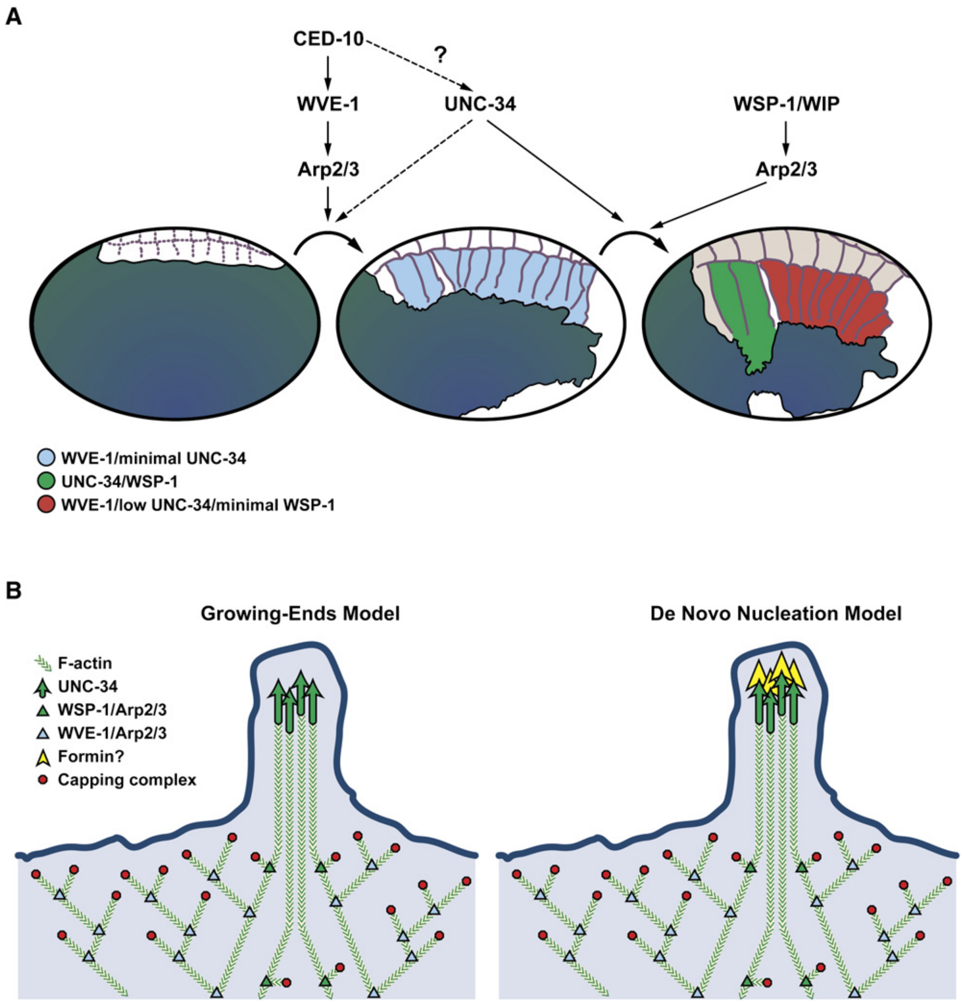

Figure S5. Models for UNC-34 and WSP-1 Action during Epithelial Migration

(A) Summary of genetic interactions during epidermal cell migration. The initiation of cell migration is largely dependent on WVE-1E activity, with partial redundancy of UNC-34, which might act downstream of CED-10/Rac (indicated by the question mark). In addition to WVE-1, leading cell migration requires UNC-34 or WSP-1/WIP activity for the proper formation of protrusions.

(B) Molecular models of UNC-34/WSP-1 synergy. In a growing-ends model, a threshold level of available barbed ends is required for filament elongation, leading to protrusion. These ends are made available by either WSP-1-mediated de novo nucleation or UNC-34-mediated barbed-end protection. Either local activity alone is sufficient to generate protrusion. In a de novo-nucleation model, a threshold level of newly created barbed ends is required. WSP-1 performs this activity by activation of the Arp2/3 complex in the same way as the previous model. UNC-34 acts primarily as a scaffolding molecule, locally recruiting a formin to nucleate new barbed ends. Again, either WSP-1 or UNC-34 local activity alone is sufficient to generate proper LCPZ morphology.

pipette to a 5% agar pad on a glass slide, submerged in M9, and sealed with a glass coverslip for filming. Embryos were filmed on a Nikon Eclipse E600 or Optiphot-2 upright microscope equipped with DIC optics and Ludl shutter controllers. Dage-MTI analog video cameras collected the data onto Macintosh G3 computers with Scion AG-5 frame grabbers. NIH Image software with custom macros was used for the compression of 3D time-lapse data into 4D QuickTime movies. Two-photon excitation microscopy was performed as previously described [S33] with a custom setup for the collection of images into Bio-Rad software, which were then compressed into 3D projected time-lapse movies with NIH Image and custom macros. Spinning-disc confocal microscopy on live GFP specimens was performed with a Yokogawa CSU10 scanhead attached to a Nikon Eclipse E600 microscope. Data were collected with a Hamamatsu ORCA-ER CCD camera wth Perkin Elmer Ultraview software on a Pentium 4 PC. Fixed specimens were viewed either with spinning-disc confocal microscopy or laser-scanning confocal microscopy with a Bio-Rad MRC1024. All live specimens were filmed at 20°C.

Quantitative Protrusion Analysis

Embryos expressing *dlg-1Δ7::gfp* [S29] were filmed with spinning-disc confocal microscopy. Twenty focal planes were captured from the ventral-most plane moving 9.5 μm into the specimen in 0.5 μm intervals. Such Z stacks were taken every 50 s throughout morphogenesis. Stacks were projected into a single image and compressed into AVI movies. These movies were then viewed on a Macintosh G4 computer with ImageJ software. Movies were expanded to 200% zoom so that analysis could be facilitated. The perimeter of the LCPZ was measured at specified time points; this measurement was divided by the width of the cell bodies so that slight differences in viewing angle and differences in cell width in the mutants could be internally controlled for (Figure S4A). These data were collected over five consecutive time points (at 50 s intervals) per embryo: the time point at which the leading cell protrusions first touch their contralateral partners and the four preceding time points for the wild-type and the last five time points of productive migration toward the ventral midline for mutant embryos. For leading cell analysis, the perimeter of the protrusions on one side was traced

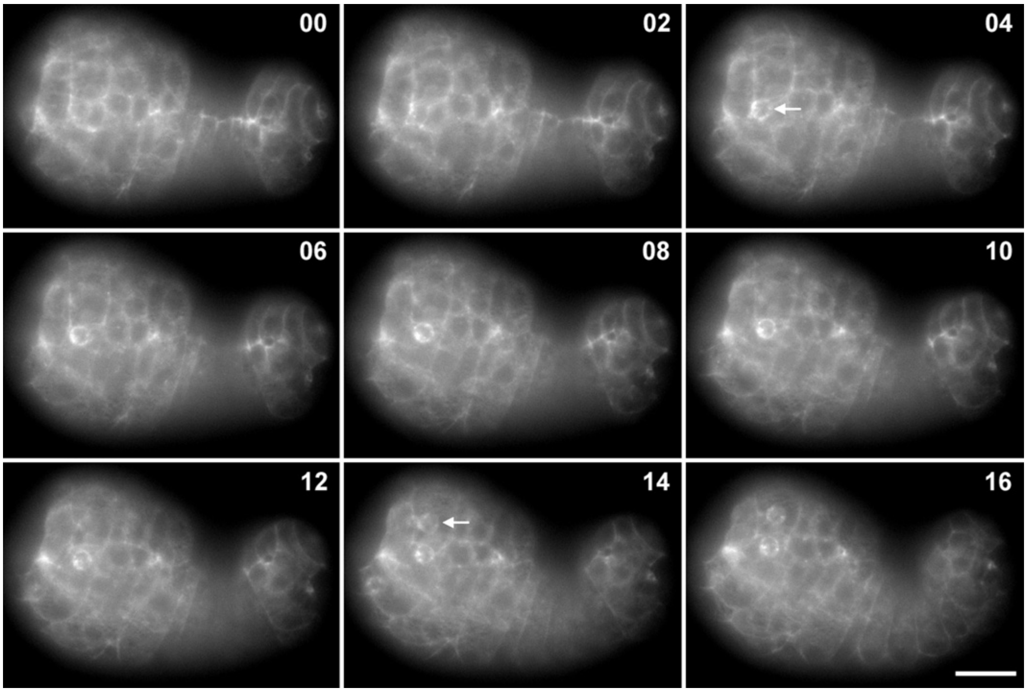

Figure S6. UNC-34::GFP Localizes Around Cell Corpses as They Are Engulfed

Ventral view of a postventral enclosure embryo at successive 2 min time points. Each frame is a Z projection of ten focal planes, encompassing 2 μm. Arrows indicate newly formed rings of UNC-34::GFP around cell corpses. Numbers indicate the time in minutes. The scale bar represents 10 μm.

in ImageJ with a Wacom Graphire3 input tablet, and the path length of the perimeter was measured. The width of the bodies of the leading cells was then measured with a segmented straight line tool, and the perimeter was divided by the width to yield an internally normalized metric. This process was repeated on the other side and again at each of the other time points in the analyzed sample. The same process was repeated for the pocket cells. Data were compared between genotypes for leading cells or pocket cells with a Wilcoxon rank sum test with $\alpha = 0.05$. A nonparametric test was chosen largely because of concerns that the population values might not be normally distributed and probably did not have equal variances; blunted mutant protrusions probably have lower variance values than do the highly dynamic wild-type protrusions. The resulting Z statistic value for leading cells was −7.085, and a similar test on pocket cells between wild-type and *unc-34(gm104)* yielded Z = −2.723, which are both well below the cutoff value for a two-tailed test with an $\alpha = 0.05$.

Antibody Staining

UNC-34 staining was performed with a modified Finney-Ruvkun [S34] protocol with 1:100 diluted polyclonal anti-UNC-34 and overnight room temperature incubations. Phalloidin staining was performed in a manner similar to previous descriptions [S19] but with fixations and incubations performed on a poly-L-lysine coated slide. Texas Red phalloidin (Molecular Probes) was applied with an overnight 4°C incubation.

Supplemental References

S1. Mullins, R.D., Heuser, J.A., and Pollard, T.D. (1998). The interaction of Arp2/3 complex with actin: nucleation, high affinity pointed end capping, and formation of branching networks of filaments. Proc. Natl. Acad. Sci. USA *95*, 6181–6186.

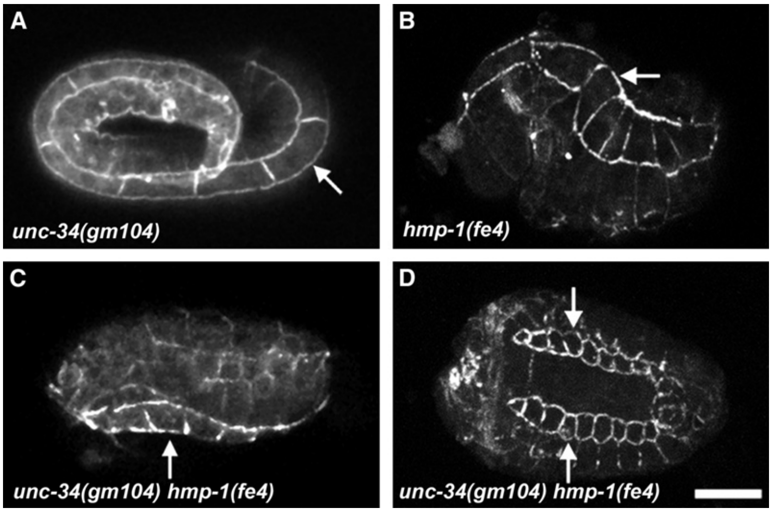

Figure S7. Junctional Localization of HMR-1/E-Cadherin Is Not Affected by the Loss of *unc-34* Function

Immunofluorescent detection of HMR-1 at epithelial junctions during embryonic elongation in *unc-34(gm104)* (A), *hmp-1(fe4)* (B), and *unc-34(gm104) hmp-1(fe4)* (C and D) embryos. (B) and (C) show a Hmp phenotype, and (D) shows a Hmr phenotype. The arrow indicates the same lateral epidermal cell (V1) in each panel to allow for comparisons between embryos (two V1 cells are visible in [D] because this is a dorsal view of the completely retracted epidermis). Anterior is to the left in all panels. The scale bar represents 10 μm.

S2. Severson, A.F., Baillie, D.L., and Bowerman, B. (2002). A Formin Homology protein and a profilin are required for cytokinesis and Arp2/3-independent assembly of cortical microfilaments in C. elegans. Curr. Biol. *12*, 2066–2075.
S3. Machesky, L.M., and Insall, R.H. (1998). Scar1 and the related Wiskott-Aldrich syndrome protein, WASP, regulate the actin cytoskeleton through the Arp2/3 complex. Curr. Biol. *8*, 1347–1356.
S4. Eden, S., Rohatgi, R., Podtelejnikov, A.V., Mann, M., and Kirschner, M.W. (2002). Mechanism of regulation of WAVE1-induced actin nucleation by Rac1 and Nck. Nature *418*, 790–793.
S5. Innocenti, M., Zucconi, A., Disanza, A., Frittoli, E., Areces, L.B., Steffen, A., Stradal, T.E., Di Fiore, P.P., Carlier, M.F., and Scita, G. (2004). Abi1 is essential for the formation and activation of a WAVE2 signalling complex. Nat. Cell Biol. *6*, 319–327.
S6. Steffen, A., Rottner, K., Ehinger, J., Innocenti, M., Scita, G., Wehland, J., and Stradal, T.E. (2004). Sra-1 and Nap1 link Rac to actin assembly driving lamellipodia formation. EMBO J. *23*, 749–759.
S7. Miki, H., Suetsugu, S., and Takenawa, T. (1998). WAVE, a novel WASP-family protein involved in actin reorganization induced by Rac. EMBO J. *17*, 6932–6941.
S8. Withee, J., Galligan, B., Hawkins, N., and Garriga, G. (2004). Caenorhabditis elegans WASP and Ena/VASP proteins play compensatory roles in morphogenesis and neuronal cell migration. Genetics *167*, 1165–1176.
S9. Soto, M.C., Qadota, H., Kasuya, K., Inoue, M., Tsuboi, D., Mello, C.C., and Kaibuchi, K. (2002). The GEX-2 and GEX-3 proteins are required for tissue morphogenesis and cell migrations in C. elegans. Genes Dev. *16*, 620–632.
S10. Lundquist, E.A., Reddien, P.W., Hartwieg, E., Horvitz, H.R., and Bargmann, C.I. (2001). Three C. elegans Rac proteins and several alternative Rac regulators control axon guidance, cell migration and apoptotic cell phagocytosis. Development *128*, 4475–4488.
S11. Blanchoin, L., Amann, K.J., Higgs, H.N., Marchand, J.B., Kaiser, D.A., and Pollard, T.D. (2000). Direct observation of dendritic actin filament networks nucleated by Arp2/3 complex and WASP/Scar proteins. Nature *404*, 1007–1011.
S12. Miki, H., Sasaki, T., Takai, Y., and Takenawa, T. (1998). Induction of filopodium formation by a WASP-related actin-depolymerizing protein N-WASP. Nature *391*, 93–96.
S13. Rohatgi, R., Ma, L., Miki, H., Lopez, M., Kirchhausen, T., Takenawa, T., and Kirschner, M.W. (1999). The interaction between N-WASP and the Arp2/3 complex links Cdc42-dependent signals to actin assembly. Cell *97*, 221–231.
S14. Anton, I.M., and Jones, G.E. (2006). WIP: A multifunctional protein involved in actin cytoskeleton regulation. Eur. J. Cell Biol. *85*, 295–304.
S15. Ramesh, N., Anton, I.M., Hartwig, J.H., and Geha, R.S. (1997). WIP, a protein associated with wiskott-aldrich syndrome protein, induces actin polymerization and redistribution in lymphoid cells. Proc. Natl. Acad. Sci. USA *94*, 14671–14676.
S16. Martinez-Quiles, N., Rohatgi, R., Anton, I.M., Medina, M., Saville, S.P., Miki, H., Yamaguchi, H., Takenawa, T., Hartwig, J.H., Geha, R.S., and Ramesh, N. (2001). WIP regulates N-WASP-mediated actin polymerization and filopodium formation. Nat. Cell Biol. *3*, 484–491.
S17. Moreau, V., Frischknecht, F., Reckmann, I., Vincentelli, R., Rabut, G., Stewart, D., and Way, M. (2000). A complex of N-WASP and WIP integrates signalling cascades that lead to actin polymerization. Nat. Cell Biol. *2*, 441–448.
S18. Sawa, M., and Takenawa, T. (2006). Caenorhabditis elegans WASP-interacting protein homologue WIP-1 is involved in morphogenesis through maintenance of WSP-1 protein levels. Biochem. Biophys. Res. Commun. *340*, 709–717.
S19. Williams-Masson, E.M., Malik, A.N., and Hardin, J. (1997). An actin-mediated two-step mechanism is required for ventral enclosure of the C. elegans hypodermis. Development *124*, 2889–2901.
S20. Sawa, M., Suetsugu, S., Sugimoto, A., Miki, H., Yamamoto, M., and Takenawa, T. (2003). Essential role of the C. elegans Arp2/3 complex in cell migration during ventral enclosure. J. Cell Sci. *116*, 1505–1518.
S21. Krause, M., Dent, E.W., Bear, J.E., Loureiro, J.J., and Gertler, F.B. (2003). Ena/VASP proteins: Regulators of the actin cytoskeleton and cell migration. Annu. Rev. Cell Dev. Biol. *19*, 541–564.
S22. Svitkina, T.M., Bulanova, E.A., Chaga, O.Y., Vignjevic, D.M., Kojima, S., Vasiliev, J.M., and Borisy, G.G. (2003). Mechanism of filopodia initiation by reorganization of a dendritic network. J. Cell Biol. *160*, 409–421.
S23. Schirenbeck, A., Arasada, R., Bretschneider, T., Stradal, T.E., Schleicher, M., and Faix, J. (2006). The bundling activity of vasodilator-stimulated phosphoprotein is required for filopodium formation. Proc. Natl. Acad. Sci. USA *103*, 7694–7699.
S24. Costa, M., Raich, W., Agbunag, C., Leung, B., Hardin, J., and Priess, J.R. (1998). A putative catenin-cadherin system mediates morphogenesis of the Caenorhabditis elegans embryo. J. Cell Biol. *141*, 297–308.
S25. Pettitt, J., Cox, E.A., Broadbent, I.D., Flett, A., and Hardin, J. (2003). The Caenorhabditis elegans p120 catenin homologue, JAC-1, modulates cadherin-catenin function during epidermal morphogenesis. J. Cell Biol. *162*, 15–22.
S26. Deng, X., Hofmann, E.R., Villanueva, A., Hobert, O., Capodieci, P., Veach, D.R., Yin, X., Campodonico, L., Glekas, A., Cordon-Cardo, C., et al. (2004). Caenorhabditis elegans ABL-1 antagonizes p53-mediated germline apoptosis after ionizing irradiation. Nat. Genet. *36*, 906–912.
S27. Koppen, M., Simske, J.S., Sims, P.A., Firestein, B.L., Hall, D.H., Radice, A.D., Rongo, C., and Hardin, J.D. (2001). Cooperative regulation of AJM-1 controls junctional integrity in Caenorhabditis elegans epithelia. Nat. Cell Biol. *3*, 983–991.
S28. Edgley, M.K., Baillie, D.L., Riddle, D.L., and Rose, A.M. (2005). Genetic balancers. In WormBook, The C. elegans Research Community, ed. http://www.wormbook.org.
S29. Firestein, B.L., and Rongo, C. (2001). DLG-1 is a MAGUK similar to SAP97 and is required for adherens junction formation. Mol. Biol. Cell *12*, 3465–3475.
S30. Walston, T., Tuskey, C., Edgar, L., Hawkins, N., Ellis, G., Bowerman, B., Wood, W., and Hardin, J. (2004). Multiple Wnt signaling pathways converge to orient the mitotic spindle in early C. elegans embryos. Dev. Cell *7*, 831–841.
S31. Kamath, R.S., Martinez-Campos, M., Zipperlen, P., Fraser, A.G., and Ahringer, J. (2001). Effectiveness of specific RNA-mediated interference through ingested double-stranded RNA in Caenorhabditis elegans. Genome Biol. *2*, 1–10.
S32. Raich, W.B., Moran, A.N., Rothman, J.H., and Hardin, J. (1998). Cytokinesis and midzone microtubule organization in Caenorhabditis elegans require the kinesin-like protein ZEN-4. Mol. Biol. Cell *9*, 2037–2049.
S33. Mohler, W.A., Simske, J.S., Williams-Masson, E.M., Hardin, J.D., and White, J.G. (1998). Dynamics and ultrastructure of developmental cell fusions in the Caenorhabditis elegans hypodermis. Curr. Biol. *8*, 1087–1090.
S34. Finney, M., and Ruvkun, G. (1990). The unc-86 gene product couples cell lineage and cell identity in C. elegans. Cell *63*, 895–905.
